# Three new species of *Pinelema* from caves in Guangxi, China (Araneae, Telemidae)

**DOI:** 10.3897/zookeys.692.11677

**Published:** 2017-08-21

**Authors:** Yang Song, Huifeng Zhao, Yufa Luo, Shuqiang Li

**Affiliations:** 1 School of Life and Environment Sciences, Gannan Normal University, Ganzhou, Jiangxi 341000, China; 2 Institute of Zoology, Chinese Academy of Sciences, Beijing 100101, China; 3 Southeast Asia Biodiversity Research Institute, Chinese Academy of Sciences, Menglun, Mengla, Yunnan 666303, China

**Keywords:** Haplogynae, karst region, SEM photographs, taxonomy, Yunnan-Guizhou Plateau

## Abstract

Three new *Pinelema* species, *P. cunfengensis* Zhao & Li, **sp. n.** (♂♀), *P. podiensis* Zhao & Li, **sp. n.** (♂♀), and *P. qingfengensis* Zhao & Li, **sp. n.** (♂♀), are described from the Guangxi Zhuang Autonomous Region of China, bringing the total number of *Pinelema* species to eight. All occur in Yunnan Province or the Guangxi Zhuang Autonomous Region. The male palp of Telemidae was studied for the first time using scanning electron microscope.

## Introduction

Telemidae Fage, 1913 is a small family of haplogyne spiders with nine genera and sixty-six extant species, with one questionable fossil species, *Telema moritzi* Wunderlich, 2004 (Wunderlich 2004). Thirty-seven species in three genera have been described from China (World Spider Catalog 2017), and most of these species are distributed on the Yunnan-Guizhou Plateau (Lin and Li 2010, Wang and Li 2010, 2016). Two species, *Telema cucphongensis* Lin & Li, 2009 and *Telema exiloculata* Lin & Li, 2009, were described from neighboring northern Vietnam (Lin et al. 2009).

The five described *Pinelema* Wang & Li, 2012 species are: *P. bailongensis* Wang & Li 2012, *P. curcici* Wang & Li, 2016, *P. huobaensis* Wang & Li, 2016, *P. xiushuiensis* Wang & Li, 2016, and *P. yaosaensis* Wang & Li, 2016. All are known from caves in southern China. Here, three new species of *Pinelema* are described, and the details of the male palp were examined using scanning electron microscope (SEM).

## Materials and methods

All specimens were examined and measured using a Leica M205 C stereomicroscope. The bodies, male palps, and female receptacles were photographed using an Olympus C7070 digital camera mounted on an Olympus SZX12 stereomicroscope. Images were subsequently combined using Helicon Focus version 6.7.1 image stacking software (http://www. heliconsoft.com). Further morphological details were studied under an Olympus BX41 compound light microscope. The left palps of the male were photographed with a Hitachi SU8010 Scanning Electron Microscope. Vulvae were removed and treated in lactic acid before being photographed. All measurements are reported in millimeters. Leg measurements are shown as total length (femur, patella, tibia, metatarsus, tarsus).

Abbreviations: **CA**, cymbial apophysis; **Em**, embolus; **Re**, receptacle; **Pa**, papillae; **SR**, spiral ridge; **SS**, spine-like structures.

Type specimens were preserved in 95% ethanol and deposited in the Institute of Zoology, Chinese Academy of Sciences (IZCAS), Beijing, China.

## Taxonomy

### Family Telemidae Fage, 1913

#### Genus Pinelema

Taxon classificationAnimaliaAraneaeTelemidae

Wang & Li, 2012

##### Type species.

*Pinelema bailongensis* Wang & Li, 2012 from Guangxi.

##### Diagnosis and description.

See Wang and Li (2012, 2016).

##### Composition.

*Pinelema* currently comprises eight species, including three new species described here. Five of them occur in the Guangxi Zhuang Autonomous Region: *P. bailongensis*, *P. cunfengensis* sp. n., *P. podiensis* sp. n., *P. qingfengensis* sp. n., *P. xiushuiensis*; the others are known from Yunnan Province in southern China: *P. curcici*, *P. huobaensis*, *P. yaosaensis*.

#### Pinelema bailongensis

Taxon classificationAnimaliaAraneaeTelemidae

Wang & Li, 2012

Figs 7A , 8A , 9A , 10A , 11A , 12A

Pinelema bailongensis Wang & Li, 2012: 82, figs 1–17 (♂♀).

##### Material examined.

1♂ (IZCAS), China: Guangxi Zhuang Autonomous Region: Baise Prefecture: Pingguo County, Bailong Cave, N23°19', E107°34', 111 m, 1.VIII.2009, C. Wang & Z. Yao.

##### Diagnosis.

The species is similar to *P. xiushuiensis* and can be distinguished by a kidney-shaped palpal bulb, many fine papillae at the retrolateral posterior part of the bulb (noted by arrows on Fig. 8A) and spine-like structures on the tip of the embolus (noted by arrows on Figs 8A, 10A) (the bulb is ovoid, and papillae and spine-like structures are absent in *P. xiushuiensis*).

##### Description.

Described by Wang and Li (2012). Here we add the description of the male palp: embolus long, tube shaped, outer margin distinctly protuberant, forming a spiral ridge (Figs 7A, 8A, 9A, 10A, 11A), continuing for approximately 180° around the embolus (Fig. 11A); the groove of the embolus has a cluster of spine-like structures (Figs 8A, 10A) (Wang and Li 2012: figs 1, 4); a distinct slit occurs in the groove, from the tip to the mesal part of the embolus (Figs 8A, 10A).

##### Distribution.

Known only from the type locality.

#### Pinelema cunfengensis

Taxon classificationAnimaliaAraneaeTelemidae

Zhao & Li sp. n.

http://zoobank.org/7F7662ED-985B-46CB-8AF3-D8719E7DA786

Figs 1 , 2 , 7 , 8 , 9 , 10 , 11 , 12 , 13

##### Type material.

**Holotype** ♂: China: Guangxi Zhuang Autonomous Region: Nanning Prefecture: Longan County: Cunfeng Cave, N23°12.58', E107°35.43', 115 m, 13.V.2015, Z. Chen & Y. Li. **Paratypes**: 3♂ and 5♀, same data as holotype.

##### Etymology.

The specific name refers to the type locality; adjective.

##### Diagnosis.

**Figure 1.**
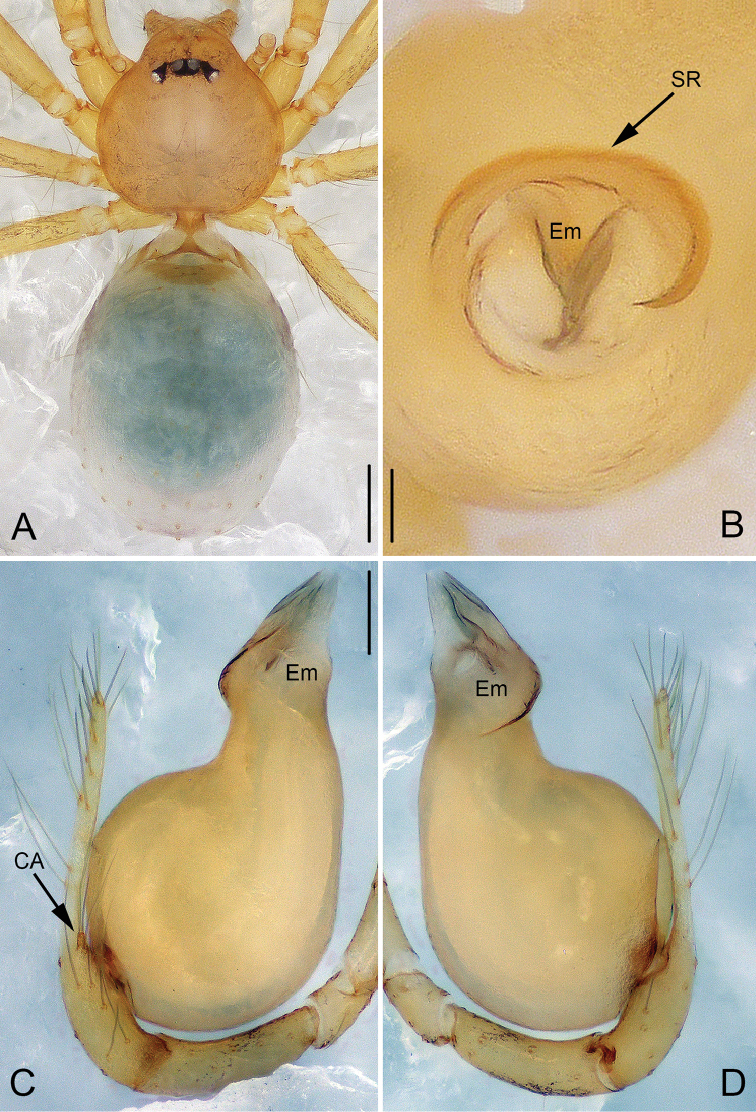
*Pinelema cunfengensis* sp. n., male holotype. **A** Habitus, dorsal view **B** Tip of embolus, apical view **C** Palp, prolateral view **D** Palp, retrolateral view. Scale bars: 0.2 mm (**A**), 0.05 mm (**B**), 0.1 mm (**C–D**).

**Figure 2.**
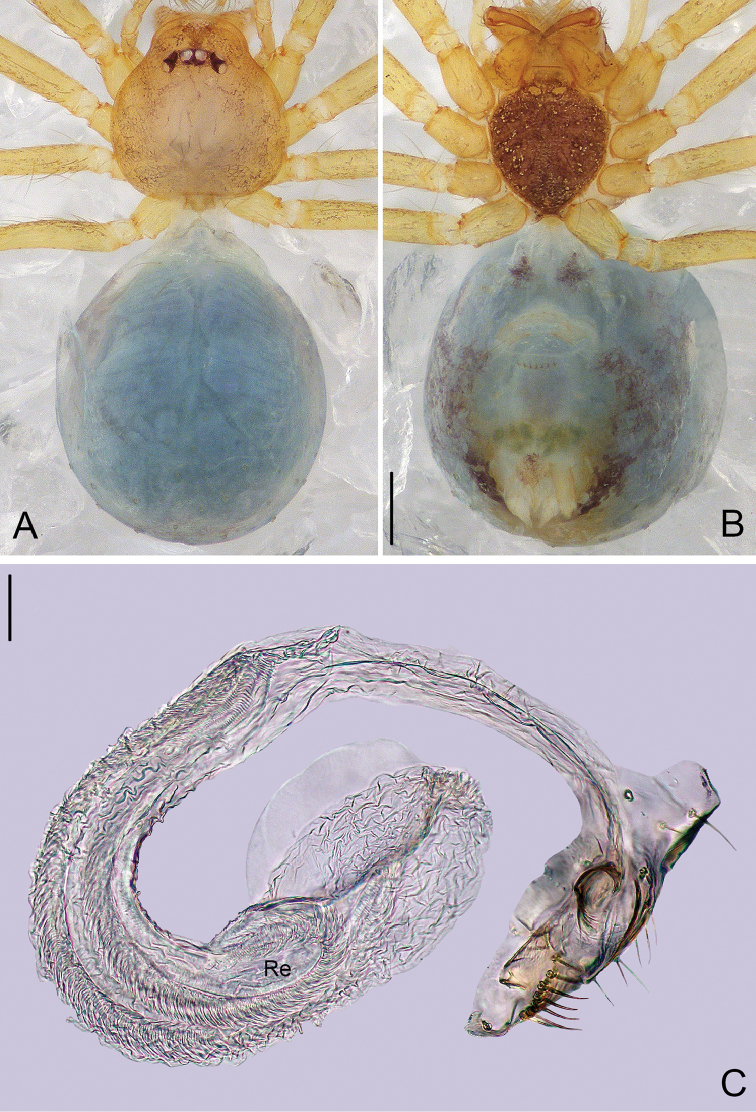
*Pinelema cunfengensis* sp. n., female paratype. **A** Habitus, dorsal view **B** Habitus, ventral view **C** Vulva, lateral view. Scale bars: 0.2 mm (**A–B**), 0.05 mm (**C**).

The new species is similar to *P. podiensis* sp. n., and can be distinguished by the well-developed eyes and greenish abdomen (Figs 1A, 2A) (eyes reduced to white spots, abdomen pale yellow in *P. podiensis* sp. n.); relatively shorter embolus (about 0.33 of bulbal length) (Figs 1C–D, 7B, 8B, 9B, 10B) (0.38 of kidney shaped bulb in *P. podiensis* sp. n.); cymbial apophysis with 3 setae (Fig. 12B) (4 setae in *P. podiensis* sp. n.); receptacle slightly curved at anterior 1/3 (Fig. 2C) (almost straight in *P. podiensis* sp. n.).

##### Description.

**Male (holotype)**: Total length 1.33. Carapace 0.50 long, 0.49 wide. Abdomen 0.79 long, 0.60 wide. Carapace dark yellow with purple-brown pattern (Fig. 1A). Six eyes, all well-developed, ringed with black. Chelicerae, legs, labium and endites dark yellow. Sternum dark brown, with sparse setae. Leg measurements: I 4.11 (1.18, 0.19, 1.33, 0.85, 0.56); II 3.59 (1.08, 0.19, 1.13, 0.69, 0.51); III 2.56 (0.81, 0.17, 0.75, 0.46, 0.36); IV 3.08 (0.99, 0.18, 0.92, 0.60, 0.38). Abdomen blue-green with purple-brown pattern ventrally.

Palp: tibia 2.5 times longer than patella, cymbium approximately two times longer than tibia, cymbial apophysis with 3 setae (Fig. 12B); bulb almost pear shaped (Figs 1C–D, 7B, 8B, 9B, 10B), the spiral ridge makes a continues approximately 180° around the embolus (Figs 1B, 11B); tip of embolus with two grooves, embolus opening slit-like, with the slit originating from the tip and terminating mesally (Figs 8B, 10B).

**Female**: Total length 1.42. Carapace 0.48 long, 0.48 wide. Abdomen 0.87 long, 0.71 wide. Coloration and pattern as in male (Figs 2A–B). Leg measurements: I 3.67 (1.08, 0.18, 1.15, 0.75, 0.51); II 3.08 (0.95, 0.16, 0.98, 0.61, 0.38); III 2.30 (0.69, 0.17, 0.65, 0.41, 0.38); IV 2.94 (0.92, 0.16, 0.89, 0.56, 0.41).

##### Distribution.

Known only from the type locality (Fig. 13).

#### Pinelema podiensis

Taxon classificationAnimaliaAraneaeTelemidae

Zhao & Li sp. n.

http://zoobank.org/E3DCFA2B-7B62-440D-AFA1-AD19A6450AB6

Figs 3 , 4 , 7 , 8 , 9 , 10 , 11 , 12 , 13

##### Type material.

**Holotype** ♂: China: Guangxi Zhuang Autonomous Region: Baise Prefecture: Debao County: Podi Cave, N23°23.51', E106°38.40', 578 m, 4.VIII.2011, C. Wang. **Paratypes**: 1♂ and 4♀, same data as holotype.

##### Etymology.

The specific name refers to the type locality; adjective.

##### Diagnosis.

**Figure 3.**
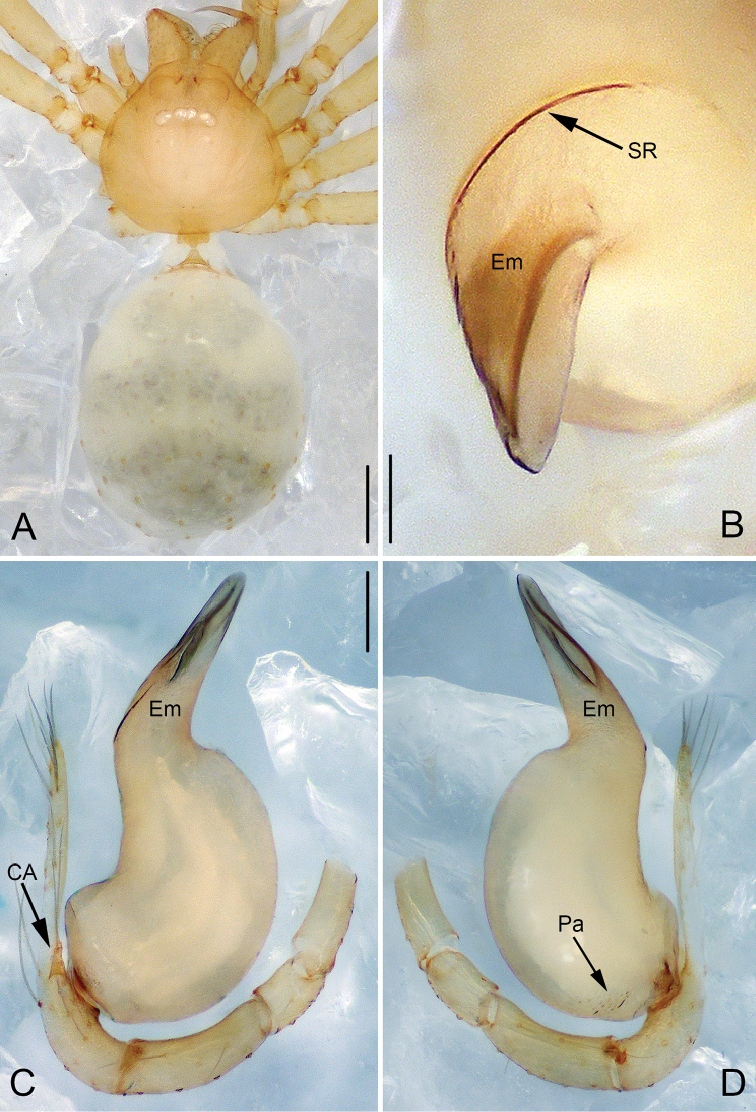
*Pinelema podiensis* sp. n., male holotype. **A** Habitus, dorsal view **B** Tip of embolus, apical view **C** Palp, prolateral view **D** Palp, retrolateral view. Scale bars: 0.2 mm (**A**), 0.05 mm (**B**), 0.1 mm (**C–D**).

**Figure 4.**
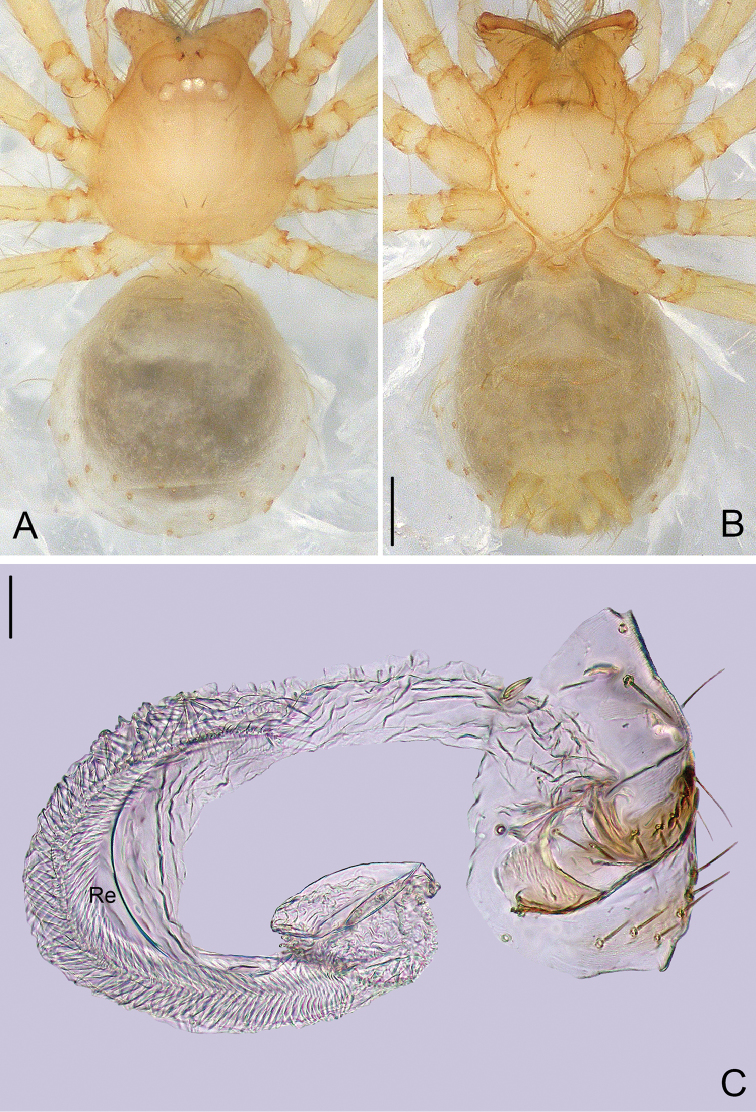
*Pinelema podiensis* sp. n., female paratype. **A** Habitus, dorsal view **B** Habitus, ventral view **C** Vulva, lateral view. Scale bars: 0.2 mm (**A–B**), 0.05 mm (**C**).

The new species is similar to *P. bailongensis*, and can be distinguished by the strongly reduced eyes and pale yellow abdomen (Figs 3A, 4A) (eyes well-developed and abdomen greenish in *P. bailongensis*); shorter embolus, about 0.38 of the bulb length (0.59 of the bulb length in *P. bailongensis*) (Figs 3C–D, 7C, 8C, 9C, 10C); receptacle coiled approximately 270° (Fig. 4C) (approximately 450° in *P. bailongensis*); the 1^st^ and 2^nd^ setae on cymbial apophysis located at the same height (Fig. 12C) (setae at different heights in *P. bailongensis*).

##### Description.

**Male (holotype)**: Total length 1.25. Carapace 0.48 long, 0.48 wide. Abdomen 0.73 long, 0.56 wide. Carapace yellow, with black yellow margins (Fig. 3A). Reduced eyes appear as white spots. Chelicerae, legs, labium and endites yellow. Sternum pale yellow, with sparse long setae. Leg measurements: I 4.35 (1.25, 0.20, 1.41, 0.89, 0.60); II 3.67 (1.06, 0.20, 1.10, 0.75, 0.56); III 2.64 (0.80, 0.20, 0.72, 0.53, 0.40); IV 3.23 (1.03, 0.19, 0.93, 0.63, 0.46). Abdomen pale yellow, with some green stripes.

Palp: tibia approximately two times longer than patella, cymbium 2.2 times longer than tibia, cymbial apophysis with 4 setae (Fig. 12C); bulb almost kidney shaped, with many fine papillae at the retrolateral posterior part of the bulb (Figs 3D, 8C); embolus long, tube shaped (Figs 3C–D, 7C, 8C, 9C, 10C); the spiral ridge continues approximately 180° around the embolus (Figs 3B, 11C); the opening of the embolus is distinct and as long as 2/3 of the embolus length (Figs 8C, 10C).

**Female**: Total length 1.45. Carapace 0.46 long, 0.45 wide. Abdomen 0.60 long, 0.60 wide. Coloration and pattern as in male (Figs 4A–B). Leg measurements: I 4.25 (1.22, 0.23, 1.34, 0.84, 0.61); II 3.59 (1.08, 0.20, 1.09, 0.71, 0.51); III 2.59 (0.83, 0.16, 0.71, 0.48, 0.41); IV 3.36 (1.10, 0.19, 0.97, 0.64, 0.46). Receptacle bent at anterior 2/3, curved approximately 270°, with a swollen sac at distal part (Fig. 4C).

##### Distribution.

Known only from the type locality (Fig. 13).

#### Pinelema qingfengensis

Taxon classificationAnimaliaAraneaeTelemidae

Zhao & Li sp. n.

http://zoobank.org/996421D3-0873-47F3-99D3-2EF4CA739C78

Figs 5 , 6 , 7 , 8 , 9 , 10 , 11 , 12 , 13

##### Type material.

**Holotype** ♂: China: Guangxi Zhuang Autonomous Region: Chongzuo Prefecture: Tiandeng County: Qingfeng Cave, N23°10.31', E107°09.38', 444 m, 26.XII.2012, Z. Chen & Z. Zhao. **Paratypes**: 1♂ and 6♀, same data as holotype.

**Figure 5.**
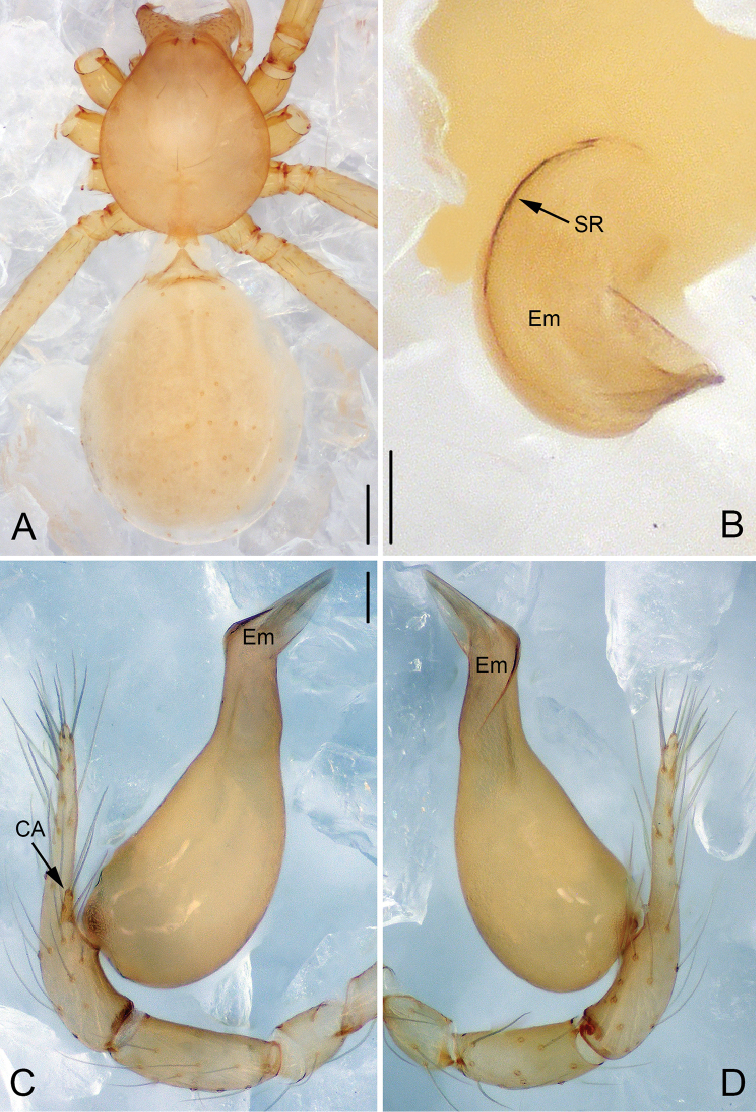
*Pinelema qingfengensis* sp. n., male holotype. **A** Habitus, dorsal view **B** Tip of embolus, apical view **C** Palp, prolateral view **D** Palp, retrolateral view. Scale bars: 0.2 mm (**A**), 0.05 mm (**B**), 0.1 mm (**C–D**).

**Figure 6.**
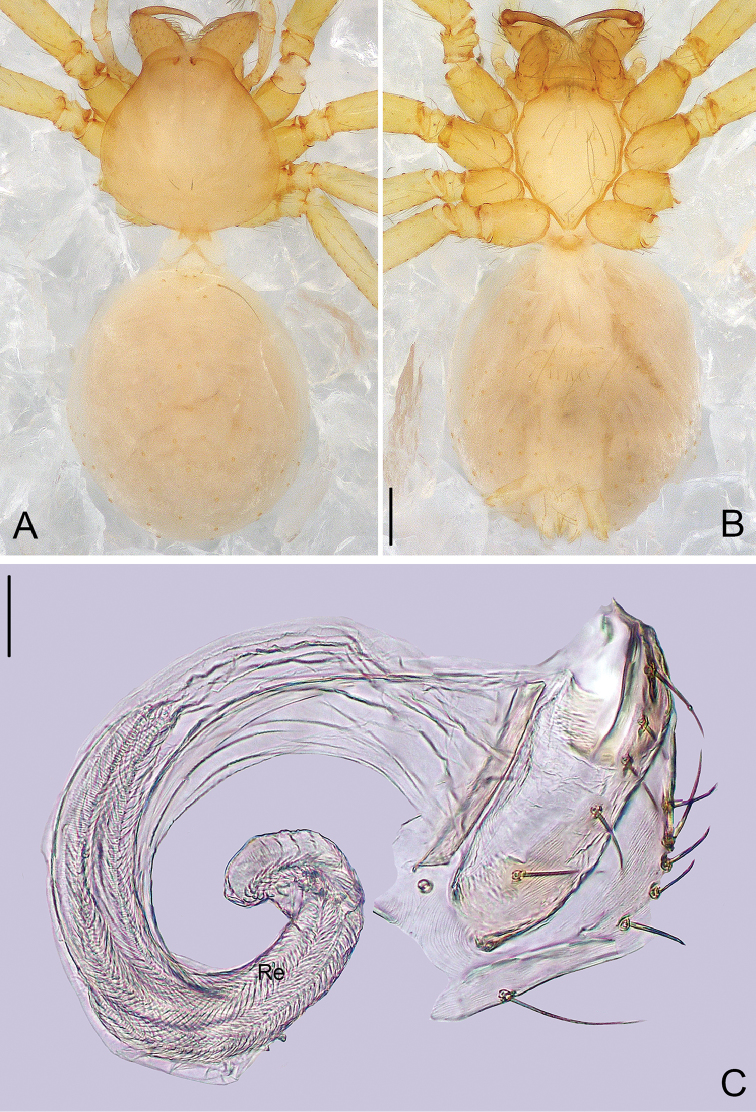
*Pinelema qingfengensis* sp. n., female paratype. **A** Habitus, dorsal view **B** Habitus, ventral view **C** Vulva, lateral view. Scale bars: 0.2 mm (**A–B**), 0.05 mm (**C**).

**Figure 7.**
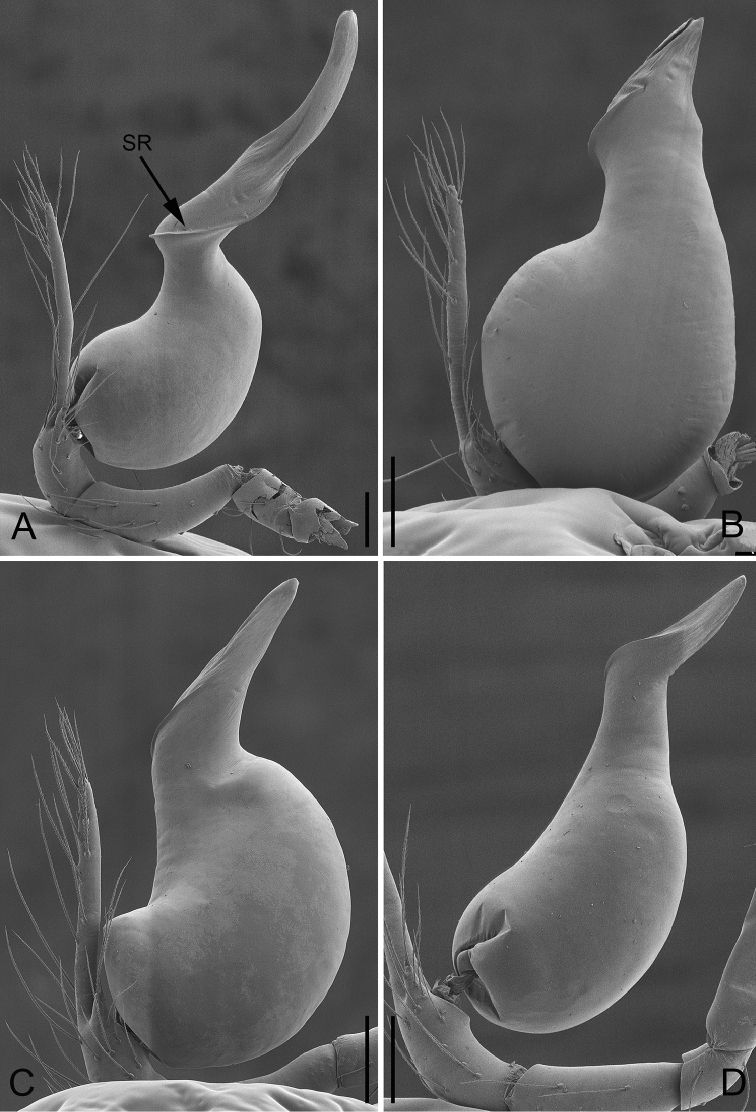
Prolateral view of male palp of *P. bailongensis* (**A**); *P. cunfengensis* sp. n. (**B**); *P. podiensis* sp. n. (**C**); *P. qingfengensis* sp. n. (**D**). Scale bars: 0.1 mm.

##### Etymology.

The specific name refers to the type locality; adjective.

##### Diagnosis.

**Figure 8.**
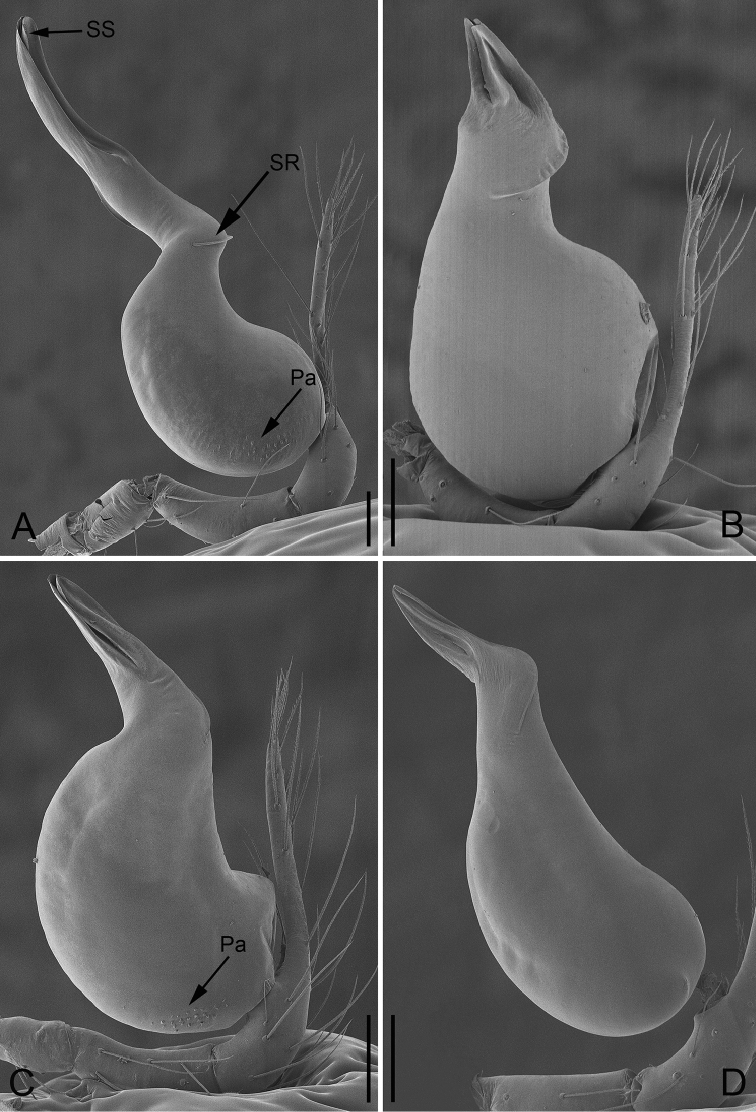
Retrolateral view of male palp of *P. bailongensis* (**A**); *P. cunfengensis* sp. n. (**B**); *P. podiensis* sp. n. (**C**); *P. qingfengensis* sp. n. (**D**). Scale bars: 0.1 mm.

**Figure 9.**
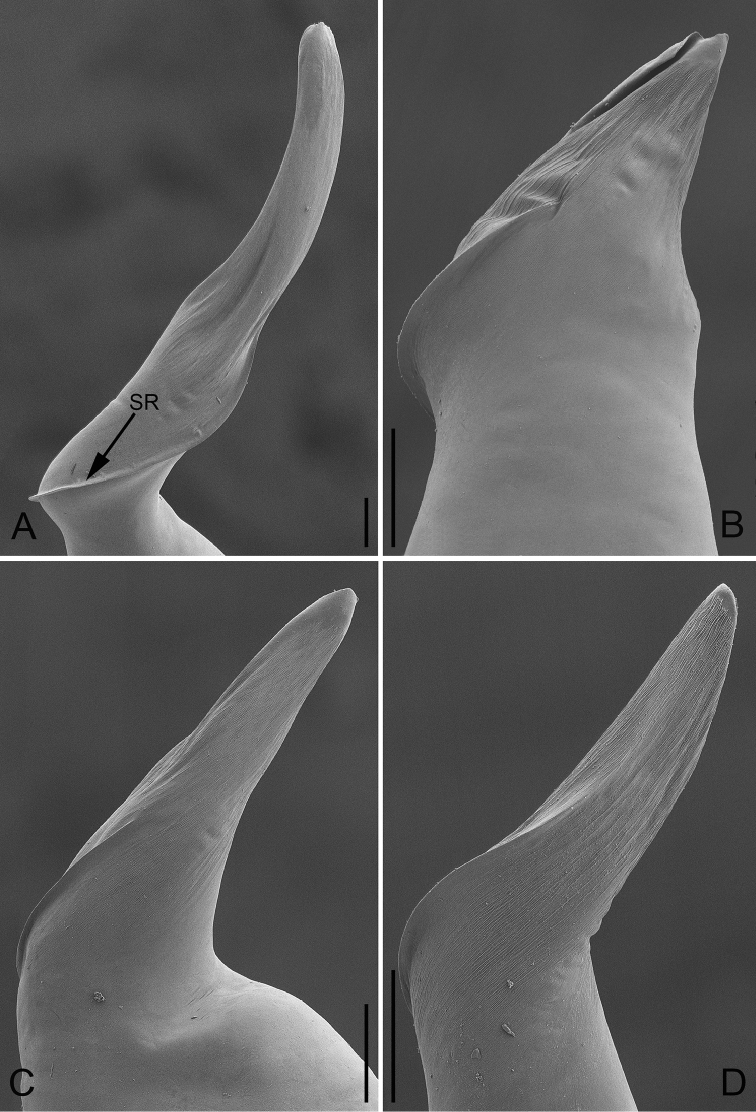
Prolateral view of embolus of *P. bailongensis* (**A**); *P. cunfengensis* sp. n. (**B**); *P. podiensis* sp. n. (**C**); *P. qingfengensis* sp. n. (**D**). Scale bars: 0.05 mm.

**Figure 10.**
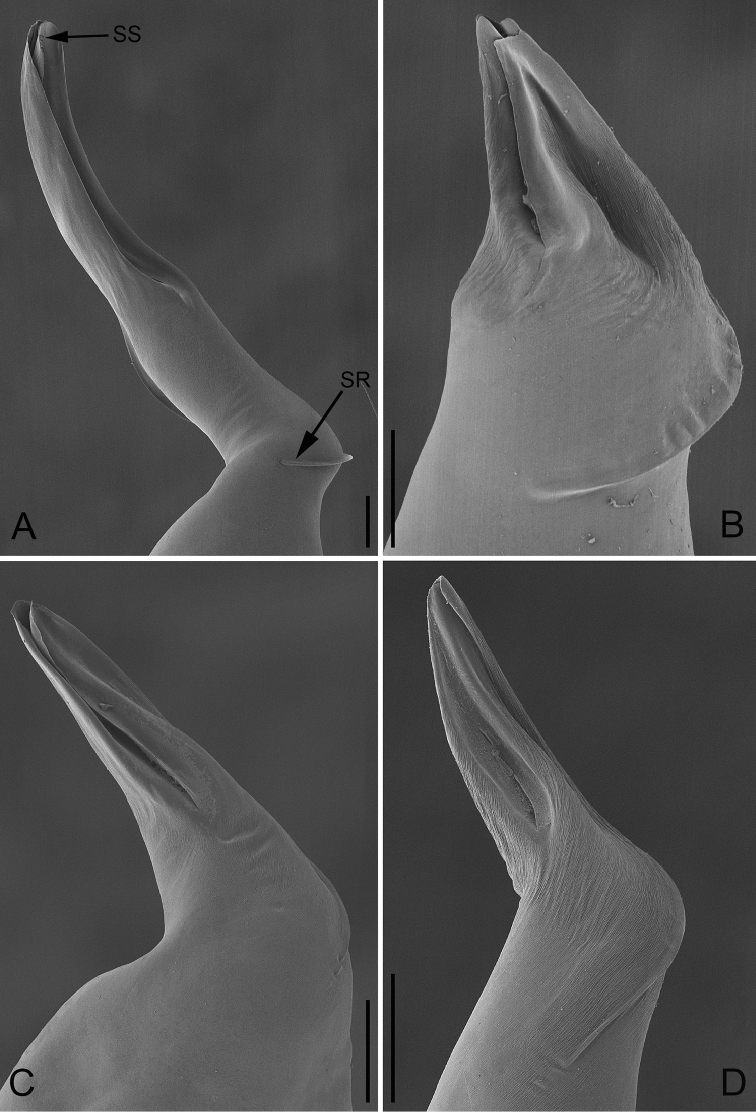
Retrolateral view of embolus of *P. bailongensis* (**A**); *P. cunfengensis* sp. n. (**B**); *P. podiensis* sp. n. (**C**); *P. qingfengensis* sp. n. (**D**). Scale bars: 0.05 mm.

The new species is similar to *P. bailongensis* and can be distinguished by the completely reduced eyes and yellow abdomen (Figs 5A, 6A) (eyes well-developed and abdomen greenish in *P. bailongensis*); the shorter embolus, 0.40 of bulb length (Figs 5C–D, 7D, 8D, 9D, 10D) (0.59 of bulb length in *P. bailongensis*); receptacle curves approximately 450° (Fig. 6C) (receptacle curves approximately 540° in *P. bailongensis*) and cymbial apophysis with five setae (Fig. 12 D) (four setae in *P. bailongensis*).

##### Description.

**Male (holotype)**: Total length 1.72. Carapace 0.71 long, 0.59 wide. Abdomen 1.00 long, 0.80 wide. Carapace yellow (Fig. 5A). Eyes entirely reduced. Chelicerae, legs, labium, and endites yellow. Sternum pale yellow. Leg measurements: I 6.53 (2.00, 0.25, 2.08, 1.44, 0.77); II 5.91 (1.76, 0.26, 1.90, 1.28, 0.71); III 4.23 (1.47, 0.23, 1.16, 0.84, 0.53); IV 4.60 (1.56, 0.23, 1.33, 0.92, 0.56). Abdomen pale yellow, with sparse long setae.

**Figure 11.**
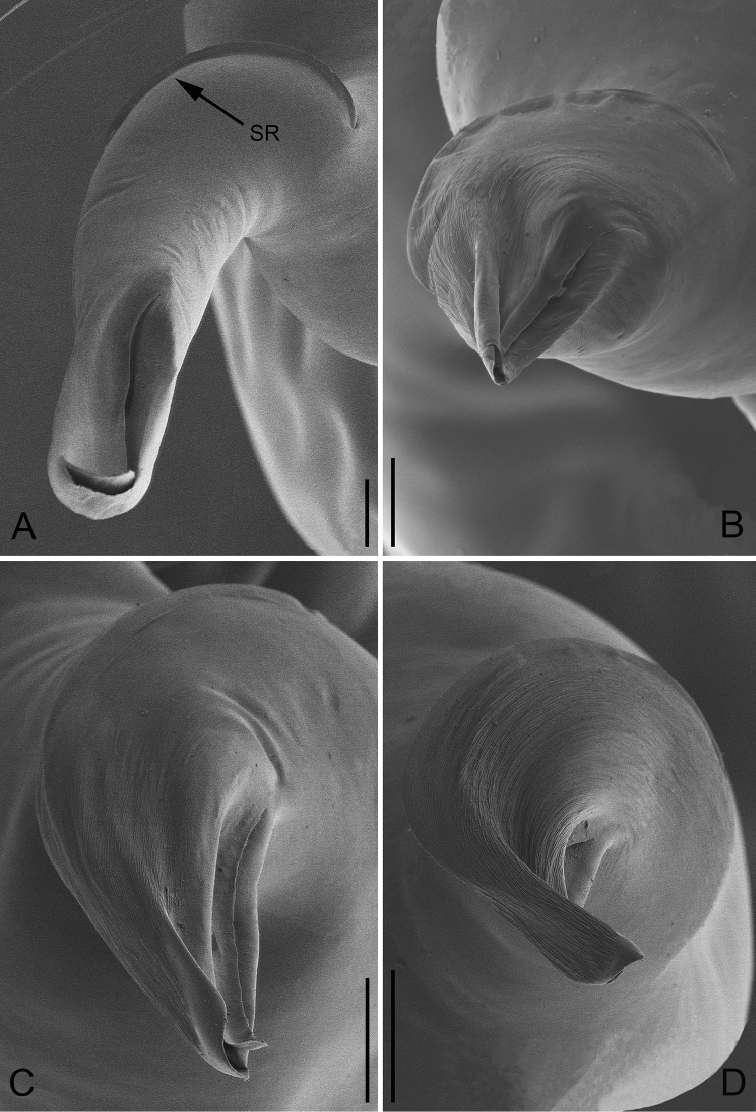
Apical view of embolus of *P. bailongensis* (**A**); *P. cunfengensis* sp. n. (**B**); *P. podiensis* sp. n. (**C**); *P. qingfengensis* sp. n. (**D**). Scale bars: 0.04 mm.

**Figure 12.**
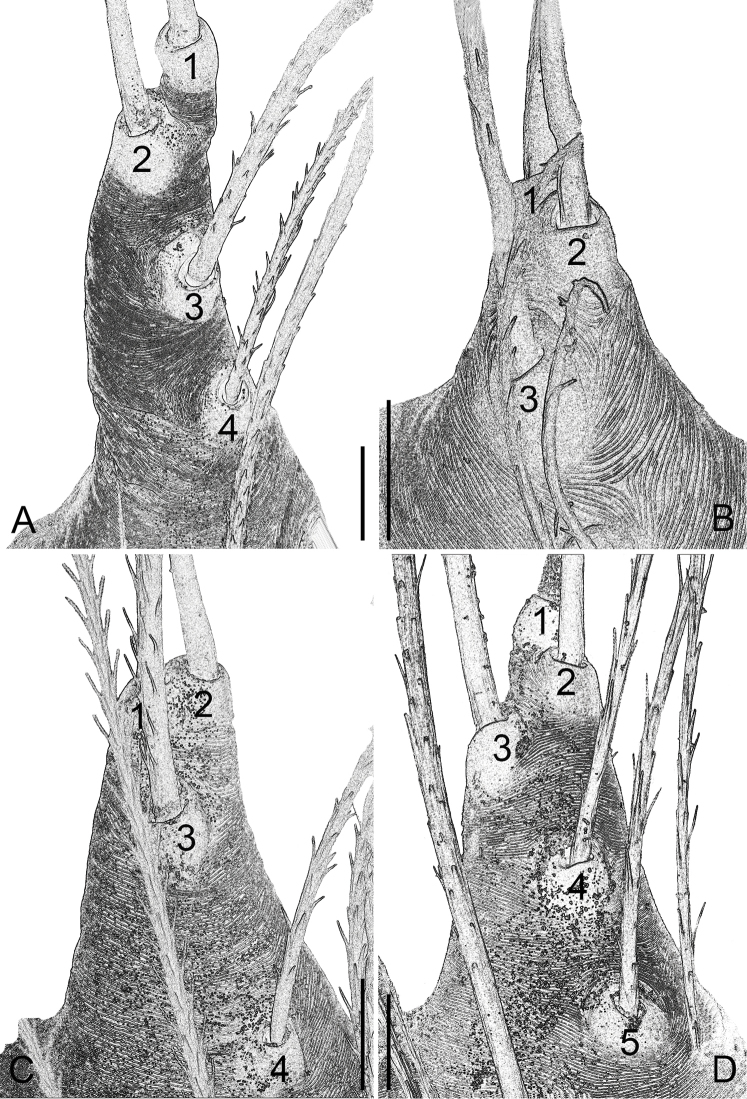
Cymbial apophysis of male palp of *P. bailongensis* (**A**); *P. cunfengensis* sp. n. (**B**); *P. podiensis* sp. n. (**C**); *P. qingfengensis* sp. n. (**D**). Scale bars: 0.01 mm. Arabic numerals refer to 1^st^–5^th^ setae. First seta in *P. podiensis* sp. n. is lost.

Palp: tibia 2.2 times longer than patella, tarsus 1.9 times longer than tibia, cymbial apophysis with five setae (Fig. 12D); bulb subconical; embolus long, tube shaped (Figs 5C–D, 7D, 8D, 9D, 10D); the spiral ridge makes a complete 360° turn around the embolus (Figs 5B, 11D), opening of embolus indistinct, as long as half length of embolus (Figs 8D, 10D).

**Female**: Total length 1.72. Carapace 0.65 long, 0.65 wide. Abdomen1.04 long, 0.89 wide. Coloration and pattern as in male (Figs 6A–B). Leg measurements: I 5.94 (2.13, 0.27, 2.13, 1.42, 0.75); II 5.23 (1.86, 0.26, 1.88, 1.24, 0.63); III 3.81 (1.41, 0.24, 1.31, 0.85, 0.51); IV 4.67 (1.72, 0.24, 1.60, 1.11, 0.59). Receptacle curved, 450° (Fig. 6C).

##### Distribution.

**Figure 13.**
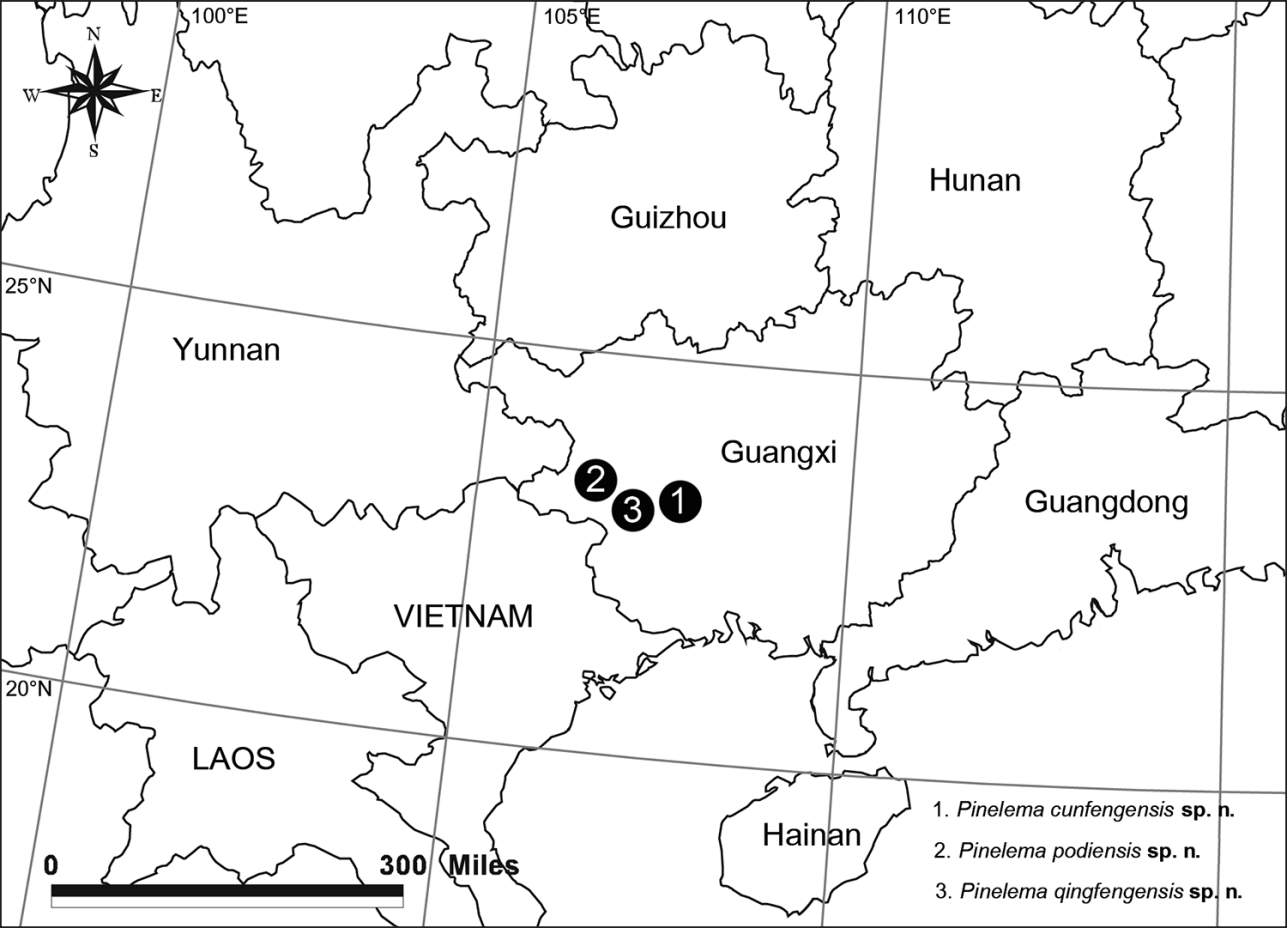
Type localities of three new *Pinelema* species from caves in Guangxi, China.

Known only from the type locality (Fig. 13).

## Supplementary Material

XML Treatment for Pinelema

XML Treatment for Pinelema bailongensis

XML Treatment for Pinelema cunfengensis

XML Treatment for Pinelema podiensis

XML Treatment for Pinelema qingfengensis
